# Editorial: The interaction of biotic and abiotic stresses

**DOI:** 10.3389/fpls.2023.1332375

**Published:** 2023-11-13

**Authors:** Prachi Pandey, Aarti Gupta, Nahla El-Sherif

**Affiliations:** ^1^Stress Interaction Lab, National Institute of Plant Genome Research, New Delhi, India; ^2^Department of Life Sciences, Pohang University of Science and Technology (POSTECH) Biotech Center, Pohang University of Science and Technology, Pohang-si, Republic of Korea; ^3^Botany Department, Faculty of Science, Ain Shams University, Cairo, Egypt

**Keywords:** abiotic and botic stresses, combined stress, stress interaction, resilience to climate change, transcriptomics, metabolomics, phytohormones, plant pathogen interaction

Plants inhabit complex environments that often encompass a myriad of abiotic and biotic stressors. Abiotic stressors, such as extreme temperatures, drought, soil salinity, high light, and flooding can profoundly influence the way pathogens and pests interact with plants. Abiotic stressors frequently serve as predisposing factors, exerting profound effects on a plant’s physiological and biochemical processes. These alterations often create conditions that are conducive to the development of diseases. For instance, prolonged exposure to high temperatures or drought can substantially compromise a plant’s natural defense mechanisms, rendering it more susceptible to fungal infections or insect infestations (Desaint et al., 2021; Sinha et al., 2021; Chilakala et al., 2022). Conversely, abiotic stressors possess the intriguing capacity to endow plants with a measure of resilience. One such response involves the activation of stress-related genes, which, in a cascading effect, bolster the plant’s resistance to biotic stressors (Yang et al., 2019). This intricate interplay between abiotic and biotic stressors underscores the dynamic nature of plant responses to environmental challenges, with both weakening and strengthening effects depending on the specific conditions and the genetic makeup of the plant. As a result, it is of paramount importance to acknowledge and thoroughly investigate the intricate interactions between these stressors when assessing their collective impact on plant health. Furthermore, it is imperative to understand that plants response to a pathogen can be different in presence or absence of another stress.

The current Research Topic delves deeply into a collection of studies that meticulously examine the influence of abiotic stressors on plant-pathogen interactions. These investigations not only unveil the intricate mechanisms underlying plant-pathogen interactions but also offer invaluable mechanistic insights into strategies for safeguarding plants against pathogens within complex environmental conditions. In a notable contribution, Chai et al. have shed light on the impact of temperature and relative humidity on the inoculum levels of *Pseudomonas amygdali* pv. *lachrymans* (Pal), [causal agent of angular leaf spot in cucumbers (*Cucumis melo*)]. Their findings paint a vivid picture of how environmental factors come into play, as high humidity and low temperatures create an environment conducive to the release and survival of pathogenic spores. These revelations underscore the critical role of environmental conditions in shaping pathogen infection dynamics and suggest a promising avenue for disease control—manipulating air temperature and humidity to mitigate disease spread.

On another front, Shahzad et al. have offered a compelling demonstration of how the shoot dieback associated with Huanglongbing (HLB) in ‘Hamlin’ sweet orange (*Citrus sinensis*) can be traced back to hypoxia stress. As the disease progresses, hypoxia is triggered, unleashing a cascade of detrimental effects. This includes the generation of excessive reactive oxygen species (ROS) that exacerbate oxidative stress, leading to cell death and ultimately resulting in shoot dieback. The authors have also elucidated the pivotal roles of plant hormones including abscisic acid, salicylic acid, and auxins in influencing infection dynamics and disease severity.

One of the most effective strategies employed to safeguard plants against fungal infections involves the application of anti-fungal compounds. However, it is of paramount importance to ascertain whether the efficacy of these anti-fungal compounds endures under extreme environmental conditions. This necessitates a profound understanding of the mechanisms by which these compounds operate. Zhang et al. delved into the intricate mechanism of action of the anti-fungal compound phenazine-1-carboxamide (PCN) against *Rhizoctonia solani* AG1IA, [causal agent of sheath blight in rice (Oryza sativa)]. Employing an integrated approach that combined transcriptomic and metabolomic analyses, the authors shed light on the remarkable mechanisms through which PCN inhibits pathogen survival inside the host plant. PCN was revealed to disrupt the very integrity of the fungal cell wall and membrane, thereby hindering the *R. solani*’s survival and proliferation. Additionally, PCN affected crucial metabolic pathways of the pathogen, including purine metabolism, ABC transporters, arachidonic acid metabolism, and phenylpropanoid biosynthesis, decreased the extracellular pH, thereby inhibiting fungal growth and invasion. Moreover, its effects extended to dampening fungal superoxide dismutase (SOD) activity, thereby impairing the pathogen’s antioxidant defense mechanisms. This multifaceted assault orchestrated by PCN exemplifies the intricate and multifarious ways in which anti-fungal compounds can subvert fungal infections.

By delving into these complex interactions between plants, pathogens and the environment researchers can develop more effective strategies for managing plant health. Notably, Adamik et al. have adeptly comprehended the complexity of combined stresses underscoring the importance of unraveling both the shared and unique responses to combined stresses. Their insights provide a remarkable understanding of the multifaceted nature of combined stresses and highlight ways for the development of superior protective strategies for plants when confronted with complex combined stresses. The authors further emphasize the need to prioritize experimental studies conducted under field or near-field conditions. This emphasis on field studies is paramount to gaining a deeper understanding of how plants adapt and respond to a multitude of stressors. It bridges the gap between controlled laboratory studies and the dynamic, complex realities faced by plants in their natural habitats.

The knowledge of combined stress induced responses and the interaction between the different stressors will empower us to implement targeted interventions, whether through breeding programs to enhance stress tolerance or by employing sustainable agricultural practices that mitigate the impact of both abiotic and biotic stressors on plants. In essence, recognizing and exploring the intricate interplay of these stressors is the key to ensuring the robust health and productivity of plants, thereby safeguarding our food supply and the overall stability of ecosystems.

## Author contributions

PP: Writing – original draft, Writing – review & editing. AG: Writing – review & editing. NE-S: Writing – review & editing.
